# Navigating the Environmental, Social, and Governance (ESG) landscape: constructing a robust and reliable scoring engine - insights into Data Source Selection, Indicator Determination, Weighting and Aggregation Techniques, and Validation Processes for Comprehensive ESG Scoring Systems

**DOI:** 10.12688/openreseurope.16278.1

**Published:** 2023-07-26

**Authors:** Yiting Liu, Joerg Osterrieder, Branka Hadji Misheva, Nicole Koenigstein, Lennart Baals

**Affiliations:** 1 Institution of Applied Data Science and Finance, Bern University of Applied Sciences, Bern Business School, Bern, Bern, 3005, Switzerland; 2 Department of High-Tech Business and Entrepreneurship, University of Twente, Enschede, Netherlands, Netherlands Antilles; 3 Nectar Digital Wealth AG, Zürich, Zürich, 8005, Switzerland

**Keywords:** Environmental, Social, and Governance (ESG); ESG Scoring Engine; ESG Indicator Selection; Weighting Methodologies; Validation

## Abstract

This white paper explores the construction of a reliable Environmental, Social, and Governance (ESG) scoring engine, with a focus on the importance of data sources and quality, selection of ESG indicators, weighting and aggregation methodologies, and the necessary validation and benchmarking procedures. The current challenges in ESG scoring and the importance of a robust ESG scoring system are addressed, citing its increasing relevance to stakeholders. Furthermore, different data types, namely self-reported data, third-party data, and alternative data, are critically evaluated for their respective merits and limitations. The paper further elucidates the complexities and implications involved in the choice of ESG indicators, illustrating the trade-offs between standardized and customized approaches. Various weighting methodologies including equal weighting, factor weighting, and multi-criteria decision analysis are dissected. The paper culminates in outlining processes for validating the ESG scoring engine, emphasizing the correlation with financial performance, and conducting robustness and sensitivity analyses. Practical examples through case studies exemplify the implementation of the discussed techniques. The white paper aims to provide insights and guidelines for practitioners, academics, and policy makers in designing and implementing robust ESG scoring systems.

## Plain language summary

This ESG white paper explores the interplay between Environmental, Social, and Governance (ESG) factors and green finance. We begin by defining ESG and green finance, exploring their evolution, and discussing their importance in financial markets. The paper emphasises the role of green finance in driving sustainable development. Next, we delve into the ESG scoring landscape. We outline various methodologies, key players in ESG ratings, and present challenges and criticisms of current ESG scoring systems. In the third section, we propose a blueprint for a reliable ESG scoring engine. This includes discussion on various data sources and the selection of ESG indicators, highlighting the role of materiality assessment, and the balance between standardized and customized indicators. We then discuss different methodologies for weighting and aggregating these indicators. The paper concludes with the necessity of validation and benchmarking of ESG scores, particularly correlating them with financial performance and performing robustness and sensitivity analyses.

## 1 Executive summary

As Environmental, Social, and Governance (ESG) scoring becomes a central component in global investment decision-making, influencing institutional investors, asset managers, and financial analysts alike, the pressing need for a reliable and consistent scoring mechanism grows evermore ( Eccles & Serafeim, 2013). As the financial sector comes to terms with the intersectionality of financial performance and sustainability, these scores play an instrumental role in shaping sustainable investment strategies and informing comprehensive risk management. However, despite the escalating significance, developing a consistent, accurate, and meaningful ESG scoring mechanism remains a formidable challenge, fraught with numerous methodological and practical hurdles ( Busch *et al*., 2016).

Navigating towards a reliable and robust ESG scoring framework is laden with complexities. One significant hurdle is the inconsistency in ESG data reporting practices across companies, with some firms neglecting to report altogether, thus undermining the quality, reliability, and comparability of data ( Chatterji *et al*., 2016). This inconsistency hampers the generation of accurate ESG scores and further compounds the problem of a lack of universally accepted, standardized ESG indicators. The absence of standardized indicators causes differing evaluations of similar aspects of ESG performance across various organizations ( Amel-Zadeh & Serafeim, 2018). In addition, the methodologies in practice for weighting and aggregating individual ESG factors into a comprehensive score often involve subjective decisions, differing by the distinct philosophies and preferences of individual ESG data providers. This variance generates discrepancies and, consequently, differing ESG scores for the same companies ( Berg *et al*., 2022). Finally, the industry wrestles with ongoing debates about the extent to which ESG scores accurately predict financial performance, with current research offering a diverse range of often contradictory findings ( Friede *et al*., 2015). These collective challenges emphasize the obstacles in the path towards achieving a reliable and consistent ESG scoring system.

In response to these challenges, this white paper presents a methodical approach to building a robust ESG scoring engine. This approach is informed by a comprehensive review of the existing literature and current best practices in the field. The proposed approach begins with improving data quality by leveraging a diverse mix of data sources, including self-reported data, third-party data, and alternative data sources. To tackle the issue of standardized ESG indicators, we propose a materiality assessment approach to identify the most relevant ESG indicators for different sectors, while also exploring the potential advantages of customized indicators for specific organizations. The paper also delves into several methodologies for weighting and aggregating ESG factors, each with their strengths and weaknesses, including equal weighting, factor weighting, and multi-criteria decision analysis (MCDA). Additionally, it explores strategies for validating and benchmarking the ESG scoring engine, including examining its correlation with financial performance and conducting robustness and sensitivity analyses ( Revelli & Viviani, 2015). By incorporating real-world case studies, this white paper aims to demonstrate the practical implementation of the proposed ESG scoring engine and its potential impact on investment decision-making. Through its systematic, research-oriented approach to overcoming the current challenges in ESG scoring, this paper provides a comprehensive guide towards creating a reliable ESG scoring engine, thereby advancing sustainable and responsible investment practices.

## 2 Introduction

As ESG considerations increasingly permeate investment decision-making on a global scale, the necessity for a reliable, consistent scoring mechanism becomes increasingly pronounced. Acknowledging the converging relevance of financial performance and sustainability, ESG scores have emerged as an instrumental tool in framing sustainable investment strategies and refining risk management practices. Despite their escalating prominence, the path towards establishing an accurate, consistent, and meaningful ESG scoring engine remains fraught with an array of methodological and practical obstacles. The present paper emerges in response to these challenges, outlining a systematic process to build a robust ESG scoring engine that can accurately reflect corporate ESG performance, contribute to sustainable investment practices, and thereby drive the transition towards a more sustainable global economy. Leveraging a comprehensive review of existing literature and best practices, this paper serves as a practical, research-driven guide to creating an effective ESG scoring engine, capable of addressing the contemporary challenges faced in the realm of ESG scoring.

### 2.1 Background of ESG scoring

The concept of ESG scoring has its roots in the broader field of corporate social responsibility (CSR) and sustainable investing. Over the last few decades, companies’ performance has come under scrutiny, not just for financial returns, but also for their impact on the environment, social welfare, and governance practices ( Clark *et al*., 2015). The genesis of ESG scoring is a reflection of the evolving expectations of stakeholders—investors, regulators, and the public—who demand a more comprehensive view of a company’s operations and performance.

At its core, ESG scoring is a method used to quantify the sustainability and societal impact of a company within these three domains: environmental, social, and governance ( Fulton *et al*., 2012). The environmental domain examines how a company manages its impact on the environment, such as through its carbon footprint, waste management, and natural resource conservation efforts. The social domain evaluates the company’s relationships with its employees, suppliers, customers, and the communities where it operates, covering areas like human rights, labor standards, and community engagement. Finally, the governance domain assesses a company’s internal system of practices, controls, and procedures, including board structure, executive compensation, and business ethics.

Originally, ESG metrics were utilized mainly by socially responsible investors who wanted to align their investments with their values ( Riedl & Smeets, 2017). However, the use of ESG scoring has since expanded dramatically, with mainstream investors recognizing that ESG factors can materially affect a company’s financial performance and risk profile ( Friede *et al*., 2015). As a result, ESG scoring has become an integral part of investment analysis and decision-making, and various methodologies have been developed to assess and compare companies’ ESG performance.

The process of ESG scoring typically involves collecting data on a wide range of ESG indicators, weighting these indicators based on their perceived importance, and then aggregating them into a single score ( Scalet & Kelly, 2010). Various ESG data providers, rating agencies, and research firms offer ESG scores and ratings, and these are increasingly being used by investors to screen investments, engage with companies, or integrate ESG factors into their portfolio construction and risk management processes ( Berg *et al*., 2022)

However, despite the growing prominence and usage of ESG scores, the field of ESG scoring is still relatively young and faces several methodological and practical challenges, which we will explore in the following sections.

### 2.2 Importance of reliable ESG scoring

The significance of reliable ESG scoring in today’s investment landscape cannot be overstated. As sustainable investing gains traction, ESG scores have emerged as vital tools for investors to evaluate companies on their sustainability performance and societal impacts. These scores are increasingly informing investment decisions, shaping company behaviors, and driving regulatory policies, underscoring their growing importance ( Eccles *et al*., 2014).

Firstly, ESG scores serve as an essential instrument for investors seeking to align their investments with their values and societal goals ( Riedl & Smeets, 2017). By providing a quantitative measure of a company’s ESG performance, these scores enable investors to differentiate companies based on their sustainability performance and to direct their investments towards those that perform well on ESG criteria. This aspect is particularly crucial given the increasing evidence that companies with strong ESG performance are likely to exhibit better long-term financial performance, lower risk profiles, and higher investor satisfaction ( Friede *et al*., 2015).

Secondly, ESG scores serve as a potent catalyst driving companies to elevate their sustainability performance and promote transparency ( Chatterji *et al*., 2016). High-quality ESG scores are an indicator of strong sustainable practices, which often lead to a host of tangible benefits for businesses. For instance, companies with superior ESG scores can secure a lower cost of capital, primarily because these scores lower the perceived risk and thus increase the firm’s attractiveness to investors ( Clark *et al*., 2015). Furthermore, improved ESG scores are often associated with enhanced brand reputation, attracting a wider customer base interested in supporting responsible businesses. In the talent market, these companies stand a better chance of attracting and retaining highly skilled employees, thereby improving productivity and promoting loyalty. Conversely, companies with inferior ESG scores may face multifaceted pressure to ameliorate their ESG practices and enhance disclosure. Such pressure could emanate from diverse stakeholders, including investors who are increasingly incorporating ESG factors into their decision-making process, regulators advocating for sustainable practices, and a public more conscious of environmental and social issues. Therefore, ESG scores not only reflect a company’s current sustainability standing but also significantly influence future sustainable practices and strategic directions.

Thirdly, reliable ESG scoring plays a pivotal role in informing and steering regulatory policies. Policymakers worldwide are increasingly capitalizing on the insights provided by ESG scores to monitor corporate behavior, refine regulatory frameworks, and incentivize sustainable business practices ( Gordon & Ringe, 2018). For instance, the European Union’s Sustainable Finance Action Plan seeks to incorporate ESG considerations into the core of the financial system, using ESG scores as one of the key determinants for investments ( Plan, 2018). Similarly, in the United States, the Securities and Exchange Commission (SEC) has been actively exploring ways to enhance the ESG disclosure requirements, leveraging ESG scores as a tool for assessing company compliance. Such regulatory emphasis on ESG scoring underscores its importance not only in shaping the corporate sustainability landscape but also in influencing broader economic and social policies. Therefore, a robust and reliable ESG scoring system serves as a critical tool for policymakers, assisting them in pushing for more sustainable corporate behaviors, developing impactful sustainable finance regulations, and ensuring their effective implementation.

### 2.3 Current challenges in ESG scoring

One of the major obstacles encountered in the ESG scoring landscape is the inconsistent practice of ESG data reporting ( Chen *et al*., 2018). The diversity in reporting across companies, coupled with the absence of stringent regulation, leads to discrepancies in the quality, reliability, and comparability of the data. Companies vary in their transparency, with some providing comprehensive data regarding their ESG practices while others selectively disclose specific factors or fail to report at all. Furthermore, the nature of disclosed data can also differ significantly, with some companies offering quantifiable metrics while others share more qualitative, narrative-based reports. These variations give rise to a lack of uniformity in the available data, which can lead to biased ESG scores and impede meaningful comparisons between organizations.

Secondly, the absence of universally accepted, standardized ESG indicators adds to the complexity of generating reliable ESG scores ( Alshehhi *et al*., 2018). Although there is some consensus regarding certain core ESG indicators, such as greenhouse gas emissions or gender diversity on boards, the interpretation, measurement, and importance assigned to these indicators can differ vastly across organizations and ESG data providers. For example, while one organization might consider direct carbon emissions as a key indicator of environmental performance, another might prioritize water usage or waste production. These disparities often lead to inconsistent ESG scores that lack comparability, hindering the integration of ESG considerations into decision-making processes.

The challenge extends to the methodology of weighting and aggregating ESG factors into a holistic ESG score ( Bassen & Kovacs, 2020). The process often entails subjective decisions and can depend heavily on the specific philosophies and preferences of individual ESG data providers. Some might give equal weight to environmental, social, and governance factors, while others may prioritize one aspect over the others based on the sector or regional context. This leads to a situation where the same company could receive drastically different ESG scores from different providers. Such discrepancies can create confusion among stakeholders and may lead to suboptimal investment and regulatory decisions.

Lastly, there is ongoing debate and inconsistency in the research findings regarding the correlation between ESG scores and financial performance ( Clark *et al*., 2015). While some studies have identified a positive correlation, suggesting that companies with higher ESG scores often exhibit better financial performance, others have found no significant relationship or even a negative correlation. Furthermore, the temporal dynamics of this relationship remain unclear, with conflicting evidence regarding the short-term and long-term effects of ESG performance on financial outcomes. This lack of clarity can deter investors and other stakeholders from fully relying on ESG scores for decision-making and can limit the impact of ESG scoring on promoting sustainable business practices.

## 3 Building a reliable ESG scoring engine

Investing in the development of a reliable ESG scoring engine demands a methodical, research-driven approach that addresses the existing challenges in the ESG scoring landscape. The subsequent sections detail the steps and considerations essential to the process, beginning with a comprehensive assessment of data sources and the resulting quality of data.

### 3.1 Data sources and quality

Critical to the efficacy of the ESG scoring engine is the richness and diversity of data that it processes. The sources of ESG data can be broadly categorized into self-reported data, third-party data, and alternative data. The amalgamation of these varied data sources can offset the limitations of any individual source and contribute to a more nuanced and comprehensive understanding of a company’s ESG performance. It’s vital to be cognizant of the quality of data from each source as it significantly influences the reliability of the resultant ESG scores.

***3.1.1 Self-reported data.*** Self-reported data is a central pillar of ESG information gathering and plays an instrumental role in feeding the ESG scoring engine. As the name implies, it encapsulates data that companies voluntarily disclose through a variety of mediums like sustainability reports, annual reports, corporate websites, press releases, and other public communications. The unique value of self-reported data is in its immediacy and specificity. Companies provide a wealth of nuanced information that reflects their individual context, specific strategies, and initiatives regarding ESG matters. Furthermore, this type of data can shed light on the corporate management’s mindset, giving a sense of their commitment and approach to sustainability ( Eccles & Youmans, 2016). Self-reported data also has the advantage of being timely. As companies generate these reports, they are usually the first source of updated information about a company’s ESG activities. This can provide investors with a near-real-time view of a company’s actions, enabling them to make informed decisions promptly.

Yet, relying exclusively on self-reported data presents its own set of challenges that could potentially compromise the ESG scoring process. One of the significant obstacles is the disparity in disclosure practices across different companies. While some organizations may provide comprehensive and quantitative data encompassing a wide array of ESG indicators, others might focus only on selective issues or furnish largely qualitative, narrative-based disclosures. The absence of standardization in reporting leads to heterogeneity in data, making it challenging to compare and assess different companies using the same criteria ( Luo *et al*., 2015). Additionally, the propensity for self-serving bias is an inherent limitation of self-reported data. Companies might have the motivation to cast their ESG performance in the most favorable light, possibly resulting in an overly optimistic portrayal of their activities. Some organizations might even engage in ”greenwashing,” where they exaggerate their environmental commitments or achievements to project a more sustainable image.

Therefore, while self-reported data serves as a crucial resource, its utilization in building an ESG scoring engine necessitates careful scrutiny. Procedures need to be put in place for systematic data review, verification, and adjustment to ensure its accuracy, reliability, and representativeness. It is also beneficial to cross-verify this information with data from other sources to strike a balance between internal perspective and external assessments.

***3.1.2 Third-party data.*** Third-party data refers to information about companies’ ESG performances that are gathered by external organizations. These entities are diverse, encompassing specialized ESG rating agencies such as MSCI and Sustainalytics, non-profit organizations, and financial data providers. This type of data provides a vital, objective perspective on companies’ ESG practices and performance.

The key advantage of third-party data is the independent stance from the companies under scrutiny. These organizations do not carry the same vested interests as the companies they appraise, thereby contributing to enhanced objectivity ( Berg *et al*., 2022). Further, these bodies typically consist of teams of professionals possessing specialized knowledge and understanding of ESG issues. Their standardized procedures for collecting, verifying, and analyzing data contribute to the reliability and consistency of the assessments they produce ( El Ghoul *et al*., 2011). Third-party data also offer broad coverage across different industries and regions, facilitating comparability and benchmarking of ESG performance among companies. Additionally, they save investors and analysts the time and resources needed to gather and process ESG data independently.

Utilizing third-party data, despite its merits, is not devoid of challenges. The usage of different indicators and weighting schemes by various ESG rating agencies often results in discrepancies in ESG scores assigned to the same entity ( Berg *et al*., 2022). The cost associated with accessing third-party data might pose a considerable burden, especially on smaller investors or companies, thus creating potential barriers to entry. Further compounding this issue is the lack of transparency often exhibited in the methodologies adopted by third-party agencies, which could compromise the comprehensibility and reproducibility of their assessments ( Clark *et al.*, 2015). Additionally, the reliability of third-party data is subject to the accuracy of their databases; misinterpretations or misunderstandings of companies’ disclosures, as well as potential shortcomings in robust data collection processes, may lead to misleading ESG scores. Hence, while third-party data contributes valuable inputs towards ESG scoring, it is crucial to navigate these challenges with due diligence and a robust understanding of their contextual implications.

Given these considerations, to maximize the benefits of third-party data while mitigating its challenges, it is crucial to critically evaluate the credibility, transparency, and comprehensiveness of different third-party data providers. In addition, it is also beneficial to combine third-party data with other data sources and adjust their assessments based on company-specific situations when developing an ESG scoring engine.

***3.1.3 Alternative data.*** Alternative data, within the context of ESG scoring, represents a broad spectrum of unconventional information sources that complement traditional financial and third-party ESG reports. This non-traditional type of data offers unique insights that often evade capture by standard data sources, thereby playing an increasingly significant role in the ESG scoring process ( Eccles *et al*., 2014). These sources can encompass social media sentiment, satellite imagery, natural language processing of news articles, government reports, patent filings, and environmental sensor data, to name a few. For example, sentiments expressed on social media platforms can provide a pulse on public perception of a company’s environmental initiatives or social responsibility commitments. Similarly, satellite images have the potential to offer real-time insights into a company’s environmental impact, such as pollution levels or deforestation activities.

Alternative data sources can enhance the depth and breadth of ESG assessment by filling gaps in traditional data sources and providing a more comprehensive perspective of a company’s ESG performance ( Hughes *et al*., 2021). Furthermore, alternative data’s capacity to deliver real-time or near-real-time insights can significantly enhance the time-liness of ESG scores, allowing investors to make more informed, responsive decisions. Additionally, the use of alternative data can act as a verification layer to self-reported and third-party data, thereby improving the reliability and validity of ESG scores ( Bolton & Kacperczyk, 2021).

Despite these potential benefits, the usage of alternative data is not without complications. Capturing, processing, and interpreting such data necessitate advanced technological and analytical capabilities that may not be available or affordable for all investors or organizations. There can also be questions regarding the accuracy, dependability, and consistency of alternative data sources, particularly given their disparate nature and the potentially unregulated nature of their generation ( Hughes *et al*., 2021). Furthermore, the integration of this eclectic data with conventional data sources can be complex and resource intensive. Lastly, the use of certain alternative data sources may involve ethical and legal considerations, including privacy concerns and issues related to data ownership ( Sila & Cek, 2017). Thus, while alternative data represents an exciting frontier for enriching ESG scoring, its adoption must be approached with due diligence and careful management.

### 3.2 Selection of ESG indicators

The selection of appropriate ESG indicators is a critical component in building a reliable ESG scoring engine. The indicators chosen will directly influence the efficacy and accuracy of the resulting ESG scores, and hence need to be carefully considered. This section discusses two key aspects associated with the selection of ESG indicators: materiality assessment, which aids in identifying the most relevant ESG indicators based on industry-specific contexts and stakeholder expectations, and the deliberation between using standardized versus customized indicators, a decision that has significant implications for the comparability and specificity of ESG scores. Through exploring these two facets, we aim to provide a comprehensive understanding of the processes and considerations involved in selecting ESG indicators, thus laying the groundwork for the development of a robust ESG scoring engine.

***3.2.1 Materiality assessment.*** One of the fundamental steps in the process of constructing a reliable ESG scoring engine entails the selection of pertinent ESG indicators, a process underpinned by the principle of materiality. In the realm of ESG scoring, materiality refers to the determination of which environmental, social, and governance factors are most salient to a particular company, considering both their potential impact on the firm’s financial performance and long-term sustainability, and their importance to stakeholders ( Amel-Zadeh & Serafeim, 2018). The concept of materiality is not static; instead, it reflects evolving stakeholder expectations, regulatory changes, and sector-specific realities.

The identification and prioritization of material ESG issues, typically referred to as a materiality assessment, necessitates a comprehensive understanding of both the company’s industry-specific context and stakeholder expectations. The materiality of an ESG factor can vary greatly across industries and regions due to disparities in operating environments, regulatory landscapes, and stakeholder priorities. For instance, an energy company operating within a carbon-intensive industry may prioritize carbon emissions as a critical ESG factor, whereas corporate governance issues might take precedence in the financial services industry ( Eccles & Youmans, 2016). Implementing a materiality assessment requires a balanced mix of qualitative and quantitative analysis, supplemented by robust stakeholder engagement. To gain insights into stakeholder perspectives on material ESG issues, data can be collected via surveys, interviews, or focus groups involving diverse stakeholder groups including investors, customers, employees, and local communities. Concurrently, quantitative analysis techniques are utilized to gauge the potential financial impact of various ESG issues, thus facilitating their effective prioritization ( Goss & Roberts, 2011).

Notwithstanding its significance, the process of materiality assessment brings with it a unique set of challenges. The process is resource-intensive, requiring significant time, expertise, and financial resources. Furthermore, it is subject to inherent biases, influenced by the subjective judgement of the assessors and the relative power and influence of different stakeholder groups. As a result, it is imperative for companies to demonstrate transparency, inclusiveness, and rigor in their materiality assessment process. Only by doing so can they derive ESG scores that accurately reflect their performance on material ESG issues and resonate with the expectations of their diverse stakeholders ( Garst *et al*., 2022).

***3.2.2 Standardized vs. customized indicators.*** In the process of establishing ESG scores, a crucial choice to be made is the selection between standardized and customized ESG indicators. These indicators can be guided by a multitude of established frameworks such as those propagated by the Global Reporting Initiative (GRI), the Sustainability Accounting Standards Board (SASB), the World Economic Forum (WEF), and even those based on the United Nations’ Sustainable Development Goals (SDGs).

Standardized ESG indicators provide a uniform comparison base for diverse companies across various industries. These frameworks outline specific aspects that need to be reported and dictate how they should be represented, thereby streamlining the process and bolstering the comparative capabilities for stakeholders. For instance, GRI’s standards are universally applicable, intended to cover all organizations, regardless of their size, sector, or geographical location. On the other hand, SASB provides sector-specific standards that address unique sustainability issues faced by various industries. These standards, therefore, enable companies to disclose information that is materially significant and useful for investment decision-making ( Eccles & Krzus, 2010).

However, these standardized indicators might not capture all material ESG issues pertinent to a specific company or those operating in unique contexts. This gap is where customized ESG indicators prove to be beneficial. Customized indicators offer flexibility by considering company-specific factors, and they enable the company to underscore aspects that are uniquely crucial to its business model, industry, or regions of operation. Despite their flexibility, customized indicators present challenges regarding comparability across different firms and industries. Additionally, the creation, implementation, and maintenance of these customized indicators may require substantial resources, which could be a vital consideration for resource-constrained firms ( Hahn *et al*., 2015).

Striking a balance between standardized and customized indicators may be an optimal approach in ESG scoring. This could involve leveraging standardized indicators for broad comparability while supplementing them with customized indicators to account for unique, context-specific ESG factors. In this way, firms can accurately represent their ESG performance, while still enabling stakeholders to make meaningful comparisons across firms and sectors. This blend of standardized frameworks, like WEF sustainability goals or SDGs, with refined, customized goals allows companies to maintain alignment with global targets while addressing their specific circumstances ( Sachs, 2012; WEF, 2020).

In conclusion, the selection between standardized and customized ESG indicators is not a binary one but involves a thoughtful blend of both. Standardized indicators, such as those recommended by GRI, SASB, the WEF sustainability goals, and the SDGs, offer a robust, universally recognized framework, providing comparability across firms and sectors. However, these might not capture all pertinent ESG issues specific to a given company or industry. That’s where customized indicators come into play, offering flexibility and company-specific relevance, albeit at the cost of reduced comparability and potentially higher resource requirements. The optimal approach, therefore, may lie in a balanced combination of both types of indicators. This could involve using standardized indicators as a solid foundation and complementing them with customized ones to account for unique, context-specific factors. This approach would help companies represent their ESG performance accurately while still allowing stakeholders to make meaningful cross-firm, cross-sector comparisons.

### 3.3 Weighting and aggregation methodologies

This subsection delves into various approaches employed in ESG scoring. These methodologies play a crucial role in evaluating and consolidating ESG indicators to derive comprehensive ESG scores for companies. This subsection examines three prominent methodologies: Equal Weighting, Factor Weighting, and Multi-criteria Decision Analysis (MCDA). We also include a detailed introduction from the mathematical perspective in our Extended data ( Liu *et al*., 2023).

***3.3.1 Equal weighting.*** The equal weighting methodology, widely utilized in the realm of ESG scoring, embraces the principle of giving each selected ESG indicator an equivalent weight in the computation of the overall ESG score. This methodology is founded on the belief that all ESG factors carry equal significance in shaping a company’s sustainability performance, effectively democratizing the importance of environmental, social, and governance aspects ( Liang & Renneboog, 2017).

This equal importance allocation underscores the methodology’s simplicity and clarity, two traits that significantly contribute to the stakeholders’ comprehension of the scoring process. In this manner, the equal weighting methodology functions as a straightforward, easily digestible approach, facilitating the transparency that is often demanded in ESG reporting. Importantly, this approach circumvents potential biases that may emerge when deciding the weights of different ESG factors. By assigning equal importance to all factors, it negates the need for subjective judgments that could skew the ESG scoring towards certain indicators, thereby preserving the integrity of the scoring process.

However, despite its appealing simplicity, critics of the equal weighting methodology point out that it may fail to capture the nuanced realities of a company’s ESG performance. Not all ESG factors may hold the same relevance for every company, and the one-size-fits-all approach may lead to an oversimplification of the unique ESG contexts that different companies operate within. For example, an energy company and a financial institution may have vastly different material ESG issues; hence a carbon emission indicator might carry more weight for the former, while corporate governance may be more pertinent to the latter. Therefore, while the equal weighting approach presents a streamlined method for ESG scoring, it may not always provide the most accurate or meaningful reflection of a company’s ESG performance ( Liang & Renneboog, 2017).

Nonetheless, the equal weighting methodology remains a vital component in the arsenal of ESG scoring methodologies, offering a transparent and unbiased approach to capturing a company’s ESG performance. Its effective deployment, particularly in conjunction with other methods and strategies, contributes to the development of a more comprehensive, meaningful, and reliable ESG scoring engine.

***3.3.2 Factor weighting.*** Factor weighting methodology, as an alternative to equal weighting, assigns different weights to different ESG indicators based on their perceived relevance or importance. This approach permits the ESG scoring process to be more aligned with the specific context of the company, thereby promoting a more accurate and meaningful reflection of its sustainability performance ( Escrig-Olmedo *et al*., 2013).

In the factor weighting methodology, the weights are often determined through an exhaustive materiality assessment or expert judgment. For instance, in a sector like energy, where environmental concerns such as greenhouse gas emissions and water usage are paramount, these indicators might be assigned greater weight. Conversely, in sectors like financial services, where governance issues like executive compensation and board diversity are more critical, these indicators might be assigned more importance ( Roca & Searcy, 2012). Notably, this method captures the complex and varied nature of ESG performance across different industries and regions. By offering a tailored approach that considers the diverse material issues faced by different companies, factor weighting arguably contributes to a more robust and comprehensive understanding of a company’s ESG performance.

Indeed, while the factor weighting methodology offers a more nuanced reflection of a company’s sustainability performance, it also introduces several challenges that need to be carefully managed. The primary concern is the potential subjectivity in the determination of weights. The process of assigning relevance or significance to different ESG indicators can vary significantly depending on individual judgment or perspective, and this variability may introduce bias into the ESG scoring process. The influence of personal bias could lead to overemphasis or underemphasis of certain ESG factors, skewing the resulting scores and potentially obscuring genuine sustainability performance ( Escrig-Olmedo *et al*., 2013). Moreover, the factor weighting methodology calls for a robust materiality assessment or expert input. This requirement not only imposes a significant demand on resources, but it also necessitates specialized skills and knowledge. Companies or rating agencies may need to engage subject matter experts, conduct comprehensive materiality assessments, or invest in advanced analytics capabilities, all of which can be complex and costly to implement. Lastly, the justification and communication of assigned weights to stakeholders can present further challenges. Transparency is a critical aspect of ESG scoring, as it enhances the credibility of the scores and fosters trust among stakeholders. However, explaining the rationale behind differing weights for various ESG factors can be difficult, particularly if the weightings are determined based on subjective assessments or proprietary models. The inability to clearly articulate why certain factors are deemed more important than others may erode stakeholder trust and undermine the perceived validity of the ESG scores ( Escrig-Olmedo *et al*., 2013). Hence, it is essential to maintain clear and open communication regarding the weighting process to ensure the credibility and acceptance of the resulting ESG scores.

In sum, while the factor weighting methodology offers a potential solution to the oversimplification issue of equal weighting, its implementation should be carefully managed to mitigate potential pitfalls and maximize its benefits for ESG scoring.

***3.3.3 Multi-criteria decision analysis.*** Multi-criteria Decision Analysis (MCDA), another method commonly employed in ESG scoring, is an advanced approach that allows for the incorporation of multiple criteria, both quantitative and qualitative, into the decision-making process ( Belton & Stewart, 2002). By integrating a variety of ESG factors with differing units of measurement, MCDA provides a structured and systematic method to analyze complex sustainability issues, balance trade-offs, and derive comprehensive ESG scores.

MCDA is particularly useful when dealing with conflicting objectives and trade-offs inherent in sustainability assessments. It allows for the simultaneous consideration of environmental, social, and governance dimensions, each comprising multiple indicators that may influence each other in myriad ways. For instance, an action that improves a company’s environmental performance may negatively impact its social or economic dimensions. MCDA provides a platform to account for such complexities, enabling a holistic evaluation of corporate sustainability ( Ishizaka & Nemery, 2013). In addition, MCDA facilitates stakeholder participation in the ESG scoring process. It can incorporate stakeholder preferences, values, and priorities into the weighting and aggregation of ESG indicators. This active involvement enhances the legitimacy and acceptability of the resulting ESG scores among various stakeholder groups.

However, the implementation of MCDA in ESG scoring presents challenges. The complexity of MCDA models necessitates a high level of expertise and advanced computational resources, potentially making it less accessible for smaller organizations or those with limited resources. Also, despite enabling a more sophisticated analysis, MCDA can be less transparent due to its complex mathematical formulations, possibly affecting stakeholders’ understanding and acceptance of the resulting ESG scores ( Zopounidis & Doumpos, 2002).

### 3.4 Validation and benchmarking

The process of validating and benchmarking the chosen ESG indicators is crucial in establishing their reliability and accuracy. This section explores two main aspects of this process: validating the correlation between ESG scores and financial performance, and conducting robustness and sensitivity analyses. By investigating the link between ESG performance and financial metrics, we aim to gauge the financial relevance of the ESG scores and confirm that they provide meaningful insights into the company’s financial health and potential risks. Meanwhile, robustness and sensitivity analyses help assess the stability of the ESG scores when subjected to variations in underlying assumptions, methodologies, or data inputs. Through these processes, we aim to ensure that the ESG scoring engine produces consistent, reliable, and accurate ESG scores.

***3.4.1 Correlation with financial performance.*** Establishing the correlation between ESG scores and financial performance is a vital part of the validation and bench-marking process in building a reliable ESG scoring engine. Empirical research has indicated a positive correlation between robust ESG performance and financial performance, signifying that companies with strong ESG practices tend to exhibit better financial results over time ( Friede *et al*., 2015). The rationale behind this correlation is rooted in the view that proactive management of ESG factors can mitigate risks, drive operational efficiency, spur innovation, and enhance stakeholder relations, all of which can contribute to superior financial outcomes.

In validating and benchmarking an ESG scoring engine, it is paramount to analyze the correlation between ESG scores and various financial performance indicators. For instance, return on assets (ROA), a measure of how efficiently a company is utilizing its assets to generate profits, can be examined alongside ESG scores. If a positive correlation is observed, this might suggest that companies with higher ESG scores tend to be more effective in their use of assets, potentially because of improved operational efficiency derived from strong ESG practices. Similarly, return on equity (ROE), which signifies the profitability of a company in relation to its equity, can be correlated with ESG scores. A positive association between ROE and ESG scores might be an indication that companies that manage ESG issues effectively are more adept at generating profits for shareholders, perhaps because they are better at risk management or have stronger relationships with stakeholders. Total Shareholder Return (TSR), which is a measure of the returns that a company has provided for its shareholders, can also be compared with ESG scores. A positive correlation between ESG scores and TSR might suggest that companies with higher ESG scores tend to provide superior returns to shareholders, possibly because they are more innovative or have more sustainable business models. In addition to these financial metrics, regression analysis can be a valuable tool in the validation process. By using regression models, it’s possible to examine the statistical relationship between ESG performance and financial performance, while controlling for confounding factors. For example, a regression model might include variables for company size, industry, and geographic location, to ensure that the observed relationship between ESG scores and financial performance is not simply a result of these other factors ( Clark *et al*., 2015).

Expanding on the complexities of establishing a causal link between ESG performance and financial outcomes, it is important to bear in mind the multitude of factors that can affect this relationship. Firstly, the correlation might be affected by firm-specific characteristics such as the industry in which the firm operates. Some industries may inherently involve more ESG-related risks and opportunities than others, and thus the strength of the ESG-financial performance correlation might vary across different industries ( Revelli & Viviani, 2015). Moreover, the size of the firm can also have a bearing on the ESG-financial performance relationship. Larger companies, with more resources at their disposal, may be in a better position to invest in and manage ESG issues, which might impact their ESG scores and potentially their financial performance. Conversely, smaller companies might face more constraints in this regard, which could influence their ESG-financial performance relationship differently ( Revelli & Viviani, 2015). Furthermore, it is also pivotal to account for the potential lag effect when validating ESG scores against financial performance. The impact of ESG initiatives on financial performance may not be immediate; it could take some time for investments in ESG practices to translate into tangible financial outcomes. Consequently, when conducting the correlation analysis, it might be necessary to consider the appropriate time frame to accurately capture this lag effect ( Revelli & Viviani, 2015). Finally, the direction of causality between ESG performance and financial performance can also be an area of complexity. While it is often presumed that strong ESG performance leads to improved financial performance, the causality might also run in the opposite direction. That is, companies that are financially successful might have more resources to invest in ESG initiatives, which in turn, could lead to higher ESG scores. Thus, the relationship between ESG performance and financial performance might be bidirectional, adding another layer of complexity to the validation process ( Revelli & Viviani, 2015).

In summary, correlating ESG performance with financial performance is an important aspect of validating and bench-marking an ESG scoring engine. This correlation analysis, involving juxtaposition of ESG scores against financial metrics such as ROA, ROE, or TSR and employing regression analysis for examining the statistical relationship, serves to gauge the financial relevance of ESG scores ( Clark *et al*., 2015). However, the process is far from straightforward, given the complex causal relationship and the various confounding factors, such as firm-specific characteristics and the potential lag effect. There is also the possibility of a bidirectional causality, where financial success enables more investment in ESG initiatives, subsequently leading to higher ESG scores ( Revelli & Viviani, 2015). Therefore, while correlating ESG performance with financial performance is an important validation step, it needs to be approached with caution, considering these complexities.

***3.4.2 Robustness and sensitivity analysis.*** Robustness and sensitivity analyses constitute another key aspect of validating and benchmarking an ESG scoring engine. Essentially, these analyses aim to assess the stability of ESG scores against changes in underlying assumptions, methodologies, or input data. This can provide valuable insights into the reliability and consistency of the ESG scoring process ( Pizzol *et al*., 2017).

Robustness analysis examines whether the ESG scores remain stable when changes are made in the scoring methodologies or input data. For instance, if a scoring engine employs a factor weighting methodology, a robustness check could involve changing the weights assigned to different ESG factors and examining whether the rankings of companies based on ESG scores significantly change or not. If the rankings remain relatively stable, it indicates that the scoring process is robust. Conversely, large changes in rankings would suggest that the ESG scores are sensitive to the weights assigned to different ESG factors, raising questions about the robustness of the scoring process ( Pizzol *et al*., 2017).

Similarly, sensitivity analysis is used to evaluate the impact of uncertainty in the input data on the ESG scores. This is particularly important given the potential inconsistencies and inaccuracies in ESG data, as discussed earlier. By adjusting the input data within certain bounds and observing the consequent changes in the ESG scores, a sensitivity analysis can provide a measure of the scoring engine’s reliability in the face of data uncertainty. If the ESG scores change substantially with small changes in the input data, it suggests that the scoring process might be overly sensitive to data inaccuracies, which could undermine its reliability.

The importance of robustness and sensitivity analyses cannot be overstated in the context of ESG scoring, particularly given the intricate complexities and nuances inherent in the ESG arena. They function as an essential guardrail that upholds the integrity of the scoring process and ensures that it remains a credible and reliable tool for diverse stakeholders. At the heart of these analyses is the principle of transparency. By conducting robustness and sensitivity analyses, ESG rating agencies make evident the robustness of their scoring process against changes and uncertainties. This fosters trust among the users of ESG scores - investors, regulators, companies, and the public - by assuring them that the scores are not whimsical or arbitrary but are in fact based on a rigorous and robust analytical process. Robustness analysis, specifically, addresses concerns about potential bias and subjectivity in the scoring process. By illustrating the stability of scores against methodological changes. For instance, changes in the weighting of ESG factors or variations in the scoring algorithm. Robustness analysis demonstrates the fairness and objectivity of the scoring process. This can be instrumental in mitigating criticisms about the lack of uniformity and consistency in ESG ratings, thereby enhancing their acceptance and use. Meanwhile, sensitivity analysis is a critical tool for dealing with the uncertainty and variability inherent in ESG data. Given that ESG data comes from diverse sources - company disclosures, third-party reports, and alternative data - and that these data can be fraught with inaccuracies, inconsistencies, or missing values, sensitivity analysis can highlight the potential impacts of these data issues on the ESG scores. If the scores are found to be highly sensitive to small changes in the data, it might necessitate improvements in the data collection, verification, or imputation processes to enhance the reliability of the scores. Furthermore, robustness and sensitivity analyses can provide valuable insights for the continual refinement and improvement of the ESG scoring process. By identifying the aspects of the scoring process that are most sensitive or unstable, these analyses can direct attention to areas that might require more rigorous methodologies, better data, or more detailed considerations of context-specific factors. In this way, they serve as a feedback mechanism that helps to enhance the sophistication, precision, and relevance of ESG scoring over time.

In conclusion, robustness and sensitivity analyses are integral components of the validation process for an ESG scoring engine. They serve to assess the reliability, consistency, and stability of the scoring process, thereby ensuring that the derived ESG scores are not overly sensitive to changes in methodologies or data inaccuracies. Through these analyses, stakeholders can gain confidence in the reliability of the ESG scoring process and its resultant scores.

## 4 Case study

The implementation of a robust ESG scoring engine in an organization requires comprehensive planning, strategic decision-making, and rigorous data analysis. This section presents a detailed case study of BlackRock ( BlackRock, n.d.), a multinational investment company, which has successfully integrated an ESG scoring engine into its existing product portfolio. BlackRock’s approach serves as a practical demonstration of the principles outlined in the preceding sections of this white paper, providing valuable insights into the real-world application and challenges of ESG scoring.

BlackRock’s ESG strategy revolves around its Aladdin operating system, an end-to-end solution for investment professionals. Built on a foundation of rigorous data quality, Aladdin incorporates more than 1,200 sustainability metrics and robust financial and climate risk assessment capabilities, aligning with the principles of data sources and quality discussed in section 3.1.

Moreover, in January 2021, BlackRock has taken a minority stake in Clarity AI, an AI driven sustainability plaform, demonstrating a firm commitment to advancing the quality, consistency, and utility of BlackRock’s ESG data. This strategic investment aligns perfectly with the principles outlined in the earlier sections of this white paper.

Upon integrating Clarity AI’s capabilities into Aladdin, BlackRock enhanced its strategic selection of ESG indicators. As described in section 3.2, Clarity AI provides detailed insights into a company’s impact on the United Nations Sustainable Development Goals (UN SDGs). This innovative approach quantifies a company’s impact based on its products, services, and operations. By offering more than 60 impact metrics, Clarity AI helps users measure their contributions to specific targets and goals, in alignment with the principles discussed in section 3.2.

The partnership also enhances BlackRock’s approach to weighting and aggregating ESG metrics. Clarity AI adopts an innovative approach to these tasks, aligning with the principles of multi-criteria decision analysis outlined in section 3.3. The platform’s proprietary assessment of ESG impact integrates climate metrics, carbon footprints, and ESG impact, ensuring a comprehensive view of a company’s performance across various dimensions.

In terms of transparency and accountability, Clarity AI shines by using various metrics to assess companies’ alignment with Net Zero frameworks and report the carbon footprint of investment portfolios. These metrics comply with the greenhouse gas emissions and carbon footprint disclosure requirements set by the Task Force on Climate-related Financial Disclosures (TCFD) and other local regulations, providing a new level of transparency and accountability.

The partnership also demonstrates BlackRock’s commitment to validation and benchmarking. Clarity AI’s platform supports regulatory and client reporting, assisting investors in meeting new sustainability disclosure obligations. This commitment to transparency and accountability aligns with the principles of robust validation and benchmarking strategies discussed in section 3.4.

In conclusion, the strategic partnership between BlackRock and Clarity AI provides a compelling real-world example of the principles and approaches discussed in this white paper. By focusing on data quality, strategic selection of ESG indicators, effective weighting and aggregation of ESG metrics, and robust validation and benchmarking strategies, they illustrate a successful path toward the implementation of a comprehensive and reliable ESG scoring engine.

## 5 Conclusion

This paper has endeavored to delineate the construction of a reliable ESG scoring engine, highlighting the crucial factors to be considered while formulating an effective ESG evaluation system. The increasing importance of ESG considerations in today’s business landscape makes it imperative for companies and investors to have a comprehensive, robust, and reliable framework to assess ESG performance.

Firstly, the significance of a diversified and high-quality data pool has been underlined. This encompasses self-reported data, third-party data, and alternative data. While each data source has its strengths and limitations, their combined use can augment the comprehensiveness and reliability of ESG scores. Furthermore, the importance of alternative data is increasing, as it provides unique insights and real-time information that may not be captured by traditional data sources.

The paper also emphasizes the necessity of a well-thought-out selection of ESG indicators. Materiality assessment, which identifies and prioritizes significant ESG issues, is central to this process. Moreover, the choice between standardized and customized indicators can have substantial implications on the relevance and applicability of the ESG scores.

In terms of weighting and aggregation methodologies, various approaches, including equal weighting, factor weighting, and multi-criteria decision analysis, have been discussed. Each method has its own merits and drawbacks, and the selection of a particular methodology should align with the company’s specific circumstances and objectives.

Lastly, the paper underscores the value of validation and benchmarking, particularly the correlation with financial performance and robustness and sensitivity analysis. These elements serve to ensure the accuracy and reliability of the ESG scores and foster confidence in the scoring engine among various stakeholders.

The case studies presented provide practical illustrations of the application and benefits of a reliable ESG scoring engine. They underscore the adaptability of the ESG scoring engine to diverse industry contexts and demonstrate how a well-structured ESG scoring engine can enhance strategic decision-making, risk management, and stakeholder communication.

In conclusion, building a reliable ESG scoring engine is a complex yet worthwhile endeavor. It requires a systematic approach, innovative data solutions, careful indicator selection, thoughtful methodology design, and rigorous validation processes. Despite the challenges, a well-constructed ESG scoring engine can drive sustainable business practices, informed investment decisions, and overall value creation in the long run. As the business environment evolves and the demand for ESG information continues to grow, the importance of a reliable ESG scoring engine will only increase, making it a critical instrument in the sustainable development toolkit of businesses and investors alike.

## Data Availability

No data are associated with this article. Open Science Framework: Navigating the Environmental, Social, and Governance (ESG) landscape: constructing a robust and reliable scoring engine. https://doi.org/10.17605/OSF.IO/4EPXJ ( Liu *et al.*, 2023). Data are available under the terms of the Creative Commons Zero ”No rights reserved” data waiver (CC0 1.0 Public domain dedication).

## References

[ref-1] Alshehhi A Nobanee H Khare N : The impact of sustainability practices on corporate financial performance: Literature trends and future research potential. *Sustainability.* 2018;10(2):494. 10.3390/su10020494

[ref-2] Amel-Zadeh A Serafeim G : Why and how investors use ESG information: Evidence from a global survey. *Financial Anal J.* 2018;74(3):87–103. 10.2139/ssrn.2925310

[ref-3] Bassen A Kovács AM : Environmental, social and governance key performance indicators from a capital market perspective. Springer Fachmedien Wiesbaden,2020;809–820. 10.1007/978-3-658-16205-4_66

[ref-4] Bayoud NS Kavanagh M Slaughter G : Corporate social responsibility disclosure and corporate reputation in developing countries: the case of Libya. *J Bus Policy Res.* 2012;7(1):131–160. Reference Source

[ref-5] Belton V Stewart T : Multiple criteria decision analysis: an integrated approacha. Springer Science & Business Media,2002. 10.1007/978-1-4615-1495-4

[ref-6] Berg F Köelbel JF Rigobon R : Aggregate confusion: The divergence of ESG ratings. *Rev Financ.* 2022;26(6):1315–1344. 10.1093/rof/rfac033

[ref-7] Bolton P Kacperczyk M : Do investors care about carbon risk? *J Financ Econ.* 2021;142(2):517–549. Reference Source

[ref-8] Busch T Bauer R Orlitzky M : Sustainable development and financial markets: Old paths and new avenues. *Bus Soc.* 2016;55(3):303–329. 10.1177/0007650315570701

[ref-9] BlackRock. (n.d.). Retrieved June 23, 2023. Reference Source

[ref-10] Chatterji AK Durand R Levine DI : Do ratings of firms converge? Implications for managers, investors and strategy researchers. *Strateg Manag J.* 2016;37(8):1597–1614. 10.1002/smj.2407

[ref-11] Chen YC Hung M Wang Y : The effect of mandatory CSR disclosure on firm profitability and social externalities: Evidence from China. *J Account Econ.* 2018;65(1):169–190. 10.1016/j.jacceco.2017.11.009

[ref-14] Clarity AI. (n.d.). Retrieved June 23, 2023. Reference Source

[ref-12] Clark GL Feiner A Viehs M : From the stockholder to the stakeholder: How sustainability can drive financial outperformance.2015. Reference Source

[ref-17] Eccles RG Ioannou I Serafeim G : The impact of corporate sustainability on organizational processes and performance. *Manage Sci.* 2014;60(11):2835–2857. Reference Source

[ref-13] Eccles RG Krzus MP : One report: Integrated reporting for a sustainable strategy. John Wiley & Sons,2010. Reference Source

[ref-15] Eccles RG Serafeim G : The performance frontier: innovating for a sustainable strategy. *Harv Bus Rev.* 2013;91(5):50–6, 58, 60, 150. 23898735

[ref-16] Eccles RG Youmans T : Materiality in corporate governance: The statement of significant audiences and materiality. *J Appl Corp Finance.* 2016;28(2):39–46. Reference Source

[ref-18] El Ghoul S Guedhami O Kwok CC : Does corporate social responsibility affect the cost of capital? *J Bank Financ.* 2011;35(9):2388–2406. 10.1016/j.jbankfin.2011.02.007

[ref-19] Escrig-Olmedo E Muñoz-Torres MJ Fernández-Izquierdo MÁ : Sustainable development and the financial system: Society’s perceptions about socially responsible investing. *Bus Strategy Environ.* 2013;22(6):410–428. 10.1002/bse.1755

[ref-20] ESG Today. (n.d.). Retrieved June 23, 2023. Reference Source

[ref-21] Friede G Busch T Bassen A : ESG and financial performance: aggregated evidence from more than 2000 empirical studies. *J Sustain Finance Invest.* 2015;5(4):210–233. 10.1080/20430795.2015.1118917

[ref-22] Fulton M Kahn B Sharples C : Sustainable investing: Establishing long-term value and performance.2012. Reference Source

[ref-53] Garst J Maas K Suijs J : Materiality Assessment Is an Art, Not a Science: Selecting ESG Topics for Sustainability Reports. *California Management Review.* 2022;65(1):64–90. 10.1177/00081256221120692

[ref-23] Goss A Roberts GS : The impact of corporate social responsibility on the cost of bank loans. *J Bank Financ.* 2011;35(7):1794–1810. 10.1016/j.jbankfin.2010.12.002

[ref-24] Gordon JN Ringe WG : The Oxford handbook of corporate law and governance. Oxford University Press,2018. Reference Source

[ref-25] Hahn R Reimsbach D Schiemann F : Organizations, climate change, and transparency: Reviewing the literature on carbon disclosure. *Organ Environ.* 2015;28(1):80–102. 10.1177/1086026615575542

[ref-26] Hughes A Urban MA Wójcik D : Alternative ESG ratings: How technological innovation is reshaping sustainable investment. *Sustainability.* 2021;13(6): 3551. 10.3390/su13063551

[ref-27] Ishizaka A Nemery P : Multi-criteria decision analysis: methods and software. John Wiley & Sons,2013. Reference Source

[ref-28] Kotsantonis S Serafeim G : Four things no one will tell you about ESG data. *J Appl Corp Finance.* 2019;31(2):50–58. 10.1111/jacf.12346

[ref-29] Liang H Renneboog L : On the foundations of corporate social responsibility. *J Finance.* 2017;72(2):853–910. 10.1111/jofi.12487

[ref-30] Liu Y Osterrieder JR Misheva BH : Navigating the Environmental, Social, and Governance (ESG) landscape: constructing a robust and reliable scoring engine. [Dataset].2023. 10.17605/OSF.IO/4EPXJ PMC10568210 37840702

[ref-31] Luo X Wang H Raithel S : Corporate social performance, analyst stock recommendations, and firm future returns. *Strateg Manage J.* 2015;36(1):123–136. 10.1002/smj.2219

[ref-34] Pizzol M Laurent A Sala S : Normalisation and weighting in life cycle assessment: *quo vadis*? *Int J Life Cycle Assess.* 2017;22:853–866. 10.1007/s11367-016-1199-1

[ref-33] Plan A : Financing Sustainable Growth.European Commission. 8.3. 2018, COM 97,2018. Reference Source

[ref-35] Revelli C Viviani JL : Financial performance of socially responsible investing (SRI): what have we learned? A meta-analysis. *Bus Ethics.* 2015;24(2):158–185. 10.1111/beer.12076

[ref-50] Riedl A Smeets P : Social preferences and portfolio choice.2017. 10.2139/ssrn.2334641

[ref-54] Roca LC Searcy C : An analysis of indicators disclosed in corporate sustainability reports. *J Clean Prod.* 2012;20(1):103–118. 10.1016/j.jclepro.2011.08.002

[ref-38] Sachs JD : From millennium development goals to sustainable development goals. *Lancet.* 2012;379(9832):2206–2211. 10.1016/S0140-6736(12)60685-0 22682467

[ref-51] Scalet S Kelly TF : CSR rating agencies: What is their global impact? *J Bus Ethics.* 2010;94:69–88. 10.1007/s10551-009-0250-6

[ref-52] Sila I Cek K : The impact of environmental, social and governance dimensions of corporate social responsibility on economic performance: Australian evidence. *Procedia Comput Sci.* 2017;120:797–804. 10.1016/j.procs.2017.11.310

[ref-43] World Economic Forum: Measuring stakeholder capitalism: Towards common metrics and consistent reporting of sustainable value creation.In: World Economic Forum,2020;1–48. Reference Source

[ref-55] Zopounidis C Doumpos M : Multi–criteria decision aid in financial decision making: methodologies and literature review. *J Multi-Crit Decis Anal.* 2002;11(4–5):167–186. 10.1002/mcda.333

