# Supplementary material for: Navigating the Environmental, Social, and Governance (ESG) landscape: constructing a robust and reliable scoring engine - insights into Data Source Selection, Indicator Determination, Weighting and Aggregation Techniques, and Validation Processes for Comprehensive ESG Scoring Systems
Source: Open Res Eur. 2023 Jul 26;3:119. [Version 1] doi: 10.12688/openreseurope.16278.1 (PMC10568210; doi:10.12688/openreseurope.16278.1)
Supplement: Peer review history

# Reviewer report: Reviewer response for version 1

**DOI:** 10.21956/openreseurope.17573.r34021

**Published:** 2023-10-12

**Authors:** Roman Matkovskyy

**Affiliations:** 1 Department of Finance and Accounting, Rennes School of Business, Rennes, France

The white paper provides a thorough exploration of the main aspects of data source selection, indicator determination, and weighting and aggregation required for constructing a reliable ESG scoring engine. It examines the methodologies, real-world applications, and validation processes, offering readers both theoretical insights and practical examples. The topic of the review is discussed comprehensively within the context of current literature.

 The authors provide a comprehensive overview of three methodologies used in ESG scoring: Equal Weighting, Factor Weighting, and Multi-criteria Decision Analysis (MCDA), effectively explaining the principles behind them.

 The paper describes the validation and benchmarking processes in the context of ESG scoring. It underscores the importance of correlating ESG performance with financial performance and highlights the significance of robustness and sensitivity analyses.

 The case study section offers an examination of BlackRock's approach to integrating an ESG scoring engine, with a particular emphasis on its partnership with Clarity AI.

 The conclusion provides a brief summary of the paper's main points, emphasizing the importance of a reliable ESG scoring engine in today's business landscape.

 Overall, the white paper makes a contribution to the discourse on ESG scoring. The text is logical, well-organized, and supported by references.

 In terms of future work, the authors might consider a further distinction between methodological and practical challenges in ESG scoring. Given that the authors provided a comprehensive discussion on standardized vs. customized indicators, the further work could offer more guidance on how some companies can strike the right balance between the two.

If applicable, is the statistical analysis and its interpretation appropriate?

Not applicable

Is the study design appropriate and is the work technically sound?

Yes

Is the work clearly and accurately presented and does it engage with the current literature?

Yes

Are the conclusions drawn adequately supported by the results?

Yes

Are sufficient details of methods and analysis provided to allow replication by others?

Yes

Are all the source data and materials underlying the results available?

Yes

Reviewer Expertise:

Finance: financial markets, FinTech and sustainability.

I confirm that I have read this submission and believe that I have an appropriate level of expertise to confirm that it is of an acceptable scientific standard.

# Reviewer report: Reviewer response for version 1

**DOI:** 10.21956/openreseurope.17573.r34022

**Published:** 2023-10-12

**Authors:** Sadettin Haluk Çitçi

**Affiliations:** 1 Department of Economics, Gebze Technical University, Gebze, Turkey

The paper provides a thorough overview of the construction of a reliable ESG scoring engine, addressing critical aspects such as data quality, indicator selection, weighting methodologies, and validation procedures.

 This comprehensive approach helps the reader grasp the complexity of the topic. The paper successfully addresses the increasing importance of ESG considerations in today's business landscape, a timely and relevant topic. It acknowledges the need for a robust framework to assess ESG performance, considering the growing interest from stakeholders. Moreover, the paper acknowledges the importance of a diversified and high-quality data pool by discussing self-reported data, third-party data, and alternative data. This recognition reflects the contemporary need for well-rounded data sources in ESG evaluations. The inclusion of the case study enhances the applicability of the discussed techniques and methodologies. This practical perspective can aid practitioners in better understanding the real-world implications.

 However, there are several dimensions in which the paper can be improved. On page 4, in section 2.2, while discussing the importance of reliable ESG scoring, the paper could benefit from also addressing potential costs associated with ESG scoring for firms. Highlighting the financial implications of choosing the wrong methodology or the direct costs of data accumulation would add depth to the paper's analysis.

 Another concern is that the manuscript could expand on the competitive effects of publicly presenting ESG scores. Addressing how such presentations influence stakeholder perceptions, industry positioning, and competitive dynamics would contribute valuable insights.

 Moreover, on page 5, left column, paragraph 4, the authors mention that while some papers established a positive correlation between ESG score and financial performance, others found no significant correlation or even a negative correlation. However, on page 9, section 3.4.1, authors only write about the positive correlation of ESG with financial performance. This unclearity in the paper creates ambiguity regarding the relation between ESG score and financial performance of firms. Discussing both positive and non-significant/negative correlations, along with any findings from studies examining the causal effect of higher ESG scores on financial performance and providing the authors’ conclusions derived from the related literature, would augment the manuscript's rigor.

 In conclusion, the paper presents a valuable contribution to the ESG scoring discourse. Its comprehensive treatment of ESG scoring aspects, timely relevance, and practical insights make it a promising resource. Addressing the critiques outlined above would elevate the manuscript's thoroughness and applicability, catering to a wider range of stakeholders engaged in ESG scoring systems.

If applicable, is the statistical analysis and its interpretation appropriate?

Not applicable

Is the study design appropriate and is the work technically sound?

Yes

Is the work clearly and accurately presented and does it engage with the current literature?

Yes

Are the conclusions drawn adequately supported by the results?

Yes

Are sufficient details of methods and analysis provided to allow replication by others?

Not applicable

Are all the source data and materials underlying the results available?

No source data required

Reviewer Expertise:

Finance, behavioral economics, industrial economics

I confirm that I have read this submission and believe that I have an appropriate level of expertise to confirm that it is of an acceptable scientific standard.

# Reviewer report: Reviewer response for version 1

**DOI:** 10.21956/openreseurope.17573.r34020

**Published:** 2023-10-12

**Authors:** Ioana-Florina Coita

**Affiliations:** 1 Department of Finance and Accounting, Faculty of Economics, University of Oradea, Oradea, Romania

Thank you very much for the opportunity to review the manuscript. It is an extensive and comprehensive work on ESG scoring methods and tools for assessing risk in the financial sector. It presents a complete description of data sources, indicators, methods, tools and validation checks for buidling a reliable scoring tool in financial domain.

 My recommendation will be, taking into account the length of the manuscript, to compress it into a shorter version so that the reader grasps the focus of the paper quicker. Keeping the actual structure of the paper, it could help the reader by rephrasing some texts that repeat some ideas. For example the introductory chapters, till part 3, could be rephrased into a single chapter like Background or Introduction. 

 In summary, while the manuscript presents a valuable contribution to the understanding of ESG scoring, it could benefit from addressing the recommendations highlighted above. With revisions addressing these concerns, the paper has the potential to be a significant addition to the Journal.

If applicable, is the statistical analysis and its interpretation appropriate?

Not applicable

Is the study design appropriate and is the work technically sound?

Yes

Is the work clearly and accurately presented and does it engage with the current literature?

Yes

Are the conclusions drawn adequately supported by the results?

Yes

Are sufficient details of methods and analysis provided to allow replication by others?

Yes

Are all the source data and materials underlying the results available?

Yes

Reviewer Expertise:

Quantitative finance, behavioural finance, sentiment analysis

I confirm that I have read this submission and believe that I have an appropriate level of expertise to confirm that it is of an acceptable scientific standard.
